# A Method for Extracting Road Boundary Information from Crowdsourcing Vehicle GPS Trajectories

**DOI:** 10.3390/s18041261

**Published:** 2018-04-19

**Authors:** Wei Yang, Tinghua Ai, Wei Lu

**Affiliations:** School of Resource and Environmental Sciences, Wuhan University, 129 Luoyu Road, Wuhan 430079, China; ywgismap@whu.edu.cn (W.Y.); whuluwei@whu.edu.cn (W.L.)

**Keywords:** vehicle trajectory, road boundary, Delaunay triangulation, map construction, road maps update

## Abstract

Crowdsourcing trajectory data is an important approach for accessing and updating road information. In this paper, we present a novel approach for extracting road boundary information from crowdsourcing vehicle traces based on Delaunay triangulation (DT). First, an optimization and interpolation method is proposed to filter abnormal trace segments from raw global positioning system (GPS) traces and interpolate the optimization segments adaptively to ensure there are enough tracking points. Second, constructing the DT and the Voronoi diagram within interpolated tracking lines to calculate road boundary descriptors using the area of Voronoi cell and the length of triangle edge. Then, the road boundary detection model is established integrating the boundary descriptors and trajectory movement features (e.g., direction) by DT. Third, using the boundary detection model to detect road boundary from the DT constructed by trajectory lines, and a regional growing method based on seed polygons is proposed to extract the road boundary. Experiments were conducted using the GPS traces of taxis in Beijing, China, and the results show that the proposed method is suitable for extracting the road boundary from low-frequency GPS traces, multi-type road structures, and different time intervals. Compared with two existing methods, the automatically extracted boundary information was proved to be of higher quality.

## 1. Introduction

Real-world road networks are subject to rapid changes due to construction, accidents, and closures. Applications such as routing planning, vehicle navigation and LBS (Location Based Service) create an increasing demand for up-to-date and accurate digital maps. Traditionally, such maps are obtained by road mapping survey and remote sensing images, which is an expensive and time-consuming process [1]. Clearly, manually creating and updating maps is too costly to meet such a demand [2], and we need a technique to create maps quickly and inexpensively that reflects daily changes to road networks.

To overcome the shortcomings of traditional road data extraction methods, user-generated content (UGC) [3] is currently being used for road information extraction in two ways: one is collaborative mapping programs, such as OpenStreetMap (OSM) [3], Wikimapia. OSM is considered as one of the most successful and popular volunteered geographic information (VGI) projects and has brought free access to a wealth of geographical information [3]. During the past decade, OSM has been used as a data source supporting a wide range of services, such as mapping [4], disaster management [5], routing and navigation [6], etc. However, these volunteer-contributed data such as OSM are highly dependent on manual work of volunteers and also come with varying quality [7].

The other one is crowdsourcing trajectory, which is now a common and efficient method for updating the road network [8]. With the widespread use of GPS tracking devices for public vehicles and personal navigation assistants, large volumes of vehicle GPS trajectory data are becoming pervasively available. This new geospatial resource contains abundant information regarding road networks, traffic conditions, points of interest [9] and driving behaviors [10]. Currently, extracting high-quality road information from low-quality tracking data is a hot topic [1,2,8,9,10,11,12,13,14,15,16,17].

Unfortunately, there are many challenges for road boundary information extraction from crowdsourcing vehicle GPS trajectories, as follows:

- First, for the low-frequency GPS traces, there are abnormal trajectory segments that may pass over areas where there is no actual road due to sparse sampling [16]. For vehicle traces with a low sampling rate, there are not enough points to cover the road. Therefore, an adaptive optimization and interpolation method is proposed to solve this problem.
- Second, a road network is hierarchical (e.g., highways and local roads), non-planar (e.g., overhead crossings), and heterogeneous (e.g., a city road network is often more complicated than rural areas) [18]. In urban main road track points are intensive in traffic hub regions, while on the other hand track points are sparse in branch roads. Considering the density disparity of GPS points, it is necessary to establish the road boundary detection model though coupling the geometry features (e.g., density distribution) and movement features (e.g., direction, speed) of tracking lines.
- Third, the amount of track points generated along a road is significantly different with various time span, which increases the difficulty of high precision boundary extraction. For example, the number of track points along a road generated in one month would be much larger than that generated in one hour. And the former data set will result wider road boundary than the real one, while the later will result thinner boundary than the real one. Thus, the boundary extraction method should adaptively deal with the nonhomogeneous distribution of track points data with different time span.

In order to overcome the aforementioned challenges, a novel method for extracting road boundary information using vehicle GPS trajectory data is presented in this paper. This work is an extension of work published in the ISPRS Workshop Conference [19] and the contributions of this paper include the following:

1. An optimization and interpolation method is proposed to filter abnormal trace segments and interpolate segments adaptively to overcome vehicle trajectory sampling sparseness.
2. A road boundary detection model (RBDM) is established to detect the road boundary from low-quality vehicle GPS trajectories by constrained Delaunay triangulation (CDT) and the Voronoi diagram, through integrating geometric features and movement features of trajectory lines.
3. After Delaunay triangles are classified by the RBDM, a regional growing method based on seed triangles is proposed to extract the road boundary according to the triangle types.
4. We have conducted empirical evaluation with a real data set of taxi GPS traces in Beijing, China.

The remainder of this paper is organized as follows: Section 2 presents a review of the related work. Section 3 outlines the procedure of our road boundary extraction method. Section 4 describes the experiments on real GPS traces datasets and evaluates the proposed method. Section 5 outlines the conclusions, and directions of future research are discussed.

## 2. Related Work

The road boundary is an indispensable component in basic geographic information, and plays an important role in a variety of fields including land use mapping, public management, and road data updates. Traditionally, road boundaries are obtained from field surveying, aerial or satellite imagery, or point cloud data [20,21], which is time consuming, costly, and labor intensive. To reduce the cost of road surveying and updating, it is necessary to propose a method for extracting road boundary VGI data automatically.

At present, extracting and updating road data using collections of GPS traces is a relatively new research topic with many seminal works [22,23,24,25,26]. These works include road geometry and topology extraction [9,11,12,15,16,17,19,22] and semantic attribution data extraction [23,24,25,26,27,28,29], in terms of the form of the output data. The problem of road boundary extraction from GPS tracks is posed as one of map inference or network construction. Map inference methods can be approximately classified into five categories: clustering; trace merging; intersection linking; kernel density estimation (KDE); and geometric model. Point clustering assumes the input consists of a set of points which are then clustered in various ways, such as DBSCAN [29] or K-means [30,31], to obtain street segments which are finally connected to a street map. A trajectory segment clustering method clusters line segments based on proximity and direction to extract road segments [11,16,29]. These methods are applicable to densely sampling GPS traces, but are not robust to GPSnoises. Trace merging-based approaches extract the road lines by incrementally inserting tracks into an initially empty map involving the consolidation of trace segments based on their geometric relations and shape similarity [25,32,33]. Intersection linking methods detect road intersections first, then extract road edges by clustering, and finally connect nodes and edges to build a road map [25,34,35,36]. The three methods can only extract road centerlines, however, and cannot extract the road boundary.

The KDE-based methods treat the road data extraction as a digital image processing procedure. Raw GPS traces are rasterized to a 2D gray image first, and a global threshold is then enforced to filter the image. The skeletonization approaches, such as the Voronoi diagram, the image-processing approaches, and vector tools such as ArcScan, extract the road centerlines [37,38,39] and road polygons [37,40]. Although the KDE method can extract the road boundary, it is sensitive to density disparities and the results have low accuracy because it is difficult to select an appropriate global threshold. The grid size of the KDE method has an especially great influence on the accuracy of the results. In addition, an effective road boundary detection model is not established leading to the KDE method having difficulty extracting a high quality road boundary from low-frequency GPS traces, especially for complex road structures or a trajectory with a different time span. Computational geometry models such as the Morse theory and the Delaunay triangulation are also used to extract road centerlines and road polygons. Recently, the Morse theory and topological simplification algorithm were used for road network reconstruction and map integration in one paper [41]. Unfortunately, the method is not used for extracting the road boundary in the work. The paper [19] applied Delaunay triangulation to obtaining traffic land-use data from vehicle GPS tracks. In this paper [42], the Delaunay triangulation is applied to extract raw road polygons for map construction. Although the Delaunay triangulation method can extract a raw road boundary, it has the following drawbacks. First, all trajectory segments are interpolated to ensure enough points to extract the boundary, which would cause more error in data for not considering the abnormal segments generated by sparsely GPS sampling. Second, it is necessary to construct a road boundary detection model for detecting the boundary accurately because the method only uses the length of the triangle edge to detect the raw boundary.

The above review reveals that a more effective method for road boundary extraction from low-frequency GPS traces needs to be developed. Based on the work of [19], we proposed a novel method to detect and extract the road boundary from low-frequency vehicle GPS traces.

## 3. Methodology

**Figure 1:**
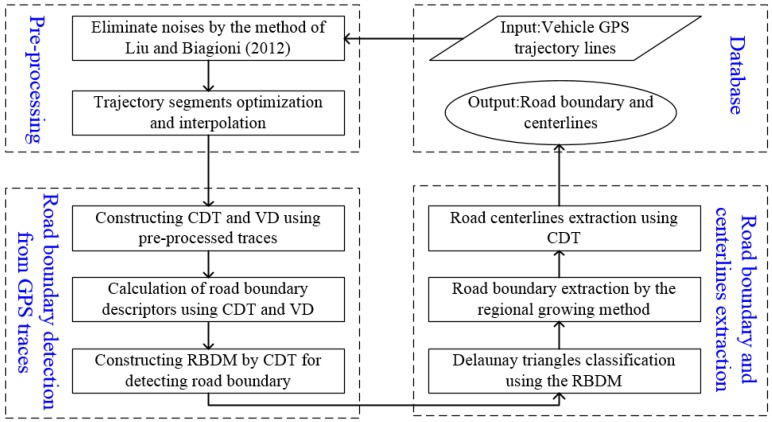
Flowchart of the proposed road boundary extraction method from GPS traces.

This section details the proposed method of extracting road boundary information (e.g., road polygons, road centerlines) from low-frequency vehicle GPS traces based on Delaunay triangulation. The flowchart is shown in Figure 1 and the approach includes three key steps:

- First, GPS traces preprocessing. After all GPS traces are preprocessed, abnormal trajectory segments are filtered and normal segments are interpolated adaptively with the optimization and interpolation method.
- Second, road boundary detection. A road boundary detection model is constructed by constraint Delaunay triangulation (CDT) and the Voronoi diagram through integrating boundary descriptors and movement features into CDT. Then, Delaunay edges are classified by the RBDM for detecting the road boundary.
- Third, road boundary and centerlines extraction. Based on seeds triangles, the road boundary is extracted by the regional growing method after using RBDM to classify Delaunay triangles, and then road centerlines are extracted from the extracted boundary by the CDT method [42].

### 3.1. Trajectory Segments Optimization and Interpolation

Trajectory noise should be removed when the trace of each taxi was acquired by linking the track points from the same car. This mainly includes conventional cleaning, track points drift, and missing. Conventional cleaning of the raw trajectory includes, for example, out of the studying area or abnormal velocity (e.g., 120 km/h) [16]. Track points drift resulting from high-rise blocks and other objects beside urban roads are smoothed by the median filter method. For the track points that are missing, these kinds of traces should be segmented and filtered [43].

**Figure 2:**
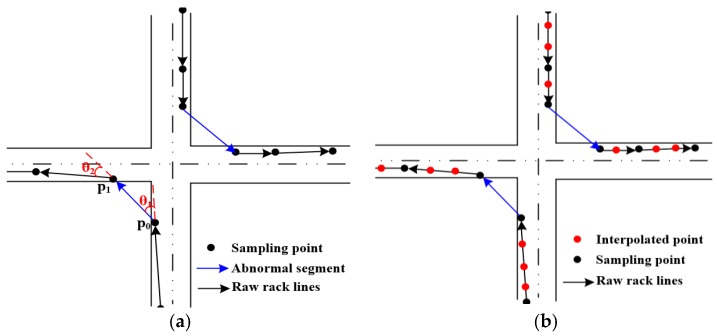
Trajectory preprocessing and trajectory segments optimization and interpolation. (**a**) Trajectory segments optimization; (**b**) segments interpolation adaptive.

Extracting the road boundary from preprocessed trace segments faces two challenges: first, there are many abnormal trace segments passing over areas where there is no actual road due to the line segment of two consecutive points being long, as shown in Figure 2a. This problem can be solved through trajectory segments optimization. For a trajectory segment, after calculating head direction angle change (it is represented by *θ*) of each track point [43], if the *θ* ∈ (45, 180°] [16], the track segment is regarded as an abnormal one (non-road trace segments) which should be filtered out, as shown in Figure 2a. Otherwise, the normal trajectory segments are optimized for constructing CDT. Second, there are not enough points to cover the road for extracting the boundary due to the vehicle tracks with low sampling rate. Therefore, the optimization trace segments are interpolated by the linear interpolation method (the step-size is 10 m in this work) illustrated in Figure 2b. This method can eliminate abnormal trace segments and ensure there are enough GPS points to cover the road.

### 3.2. Road Boundary Detection Based on Constraint Delaunay Triangulation

Extracting road boundary information from vehicle trajectories is the process of constructing a road boundary detection model using geometry features and movement features of track points to detect road boundary, which is a spatial proximity analysis problem. Delaunay triangulation is widely used in spatial proximity detection [44,45], spatial data clustering [46,47], trajectory data mining [19,23,33,42] and map generalization [48,49]. Therefore, this paper uses constraint Delaunay triangulation to detect and extract road boundary from vehicle GPS tracking lines.

#### 3.2.1. Calculation of Road Boundary Descriptors

**Figure 3:**
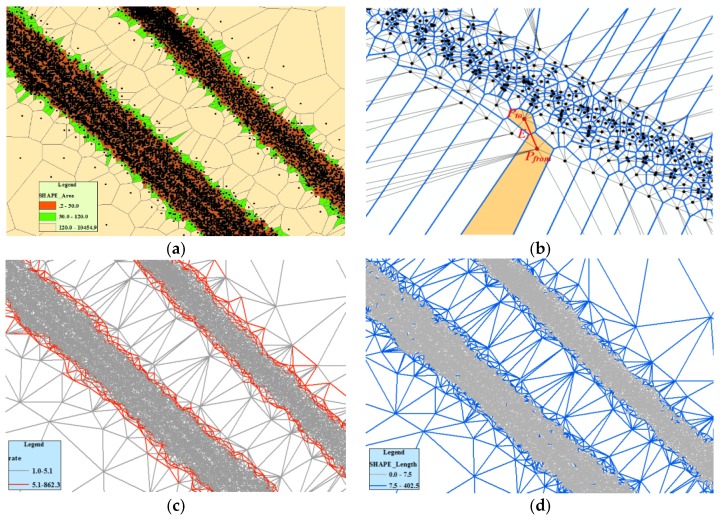
The road boundary descriptors. (**a**) Voronoi diagram constructed with track lines; (**b**) the method of calculating DCRI; (**c**) detecting the road boundary using DCRI; (**d**) detecting the road boundary using DI.

Our method is based on an assumption and observation. The assumption is that GPS traces will tend to cluster near the center of each lane with some spread, and fit a Gaussian mixture model to perpendicular cross sections of the traces across the road [24,25,26]. A large number of high-precision GPS data should be in the road region; otherwise, a small number of low-precision GPS data should be in the non-road region [23,24], as shown in Figure 3a. The observation is that the density of track points in the road area should be high, and the distance between points should be short. Instead, the density of non-road areas is low, and the distance between points is long (Figure 3a). The point density on both sides of the boundary is significantly different. Therefore, the boundary descriptors include density change rate, distance, and movement features.

First, the Density Change Rate Index is stated. Traditionally, density is described as “a measure of how much mass is obtained in a given unit volume (density = mass/volume)”. However, the unit is arbitrary and some important parameters such as the grid resolution are not easy to determine. The Voronoi diagram partitions a plane into regions by equal-area splitting based on distance to points in a set and can reflect their density distribution pattern [45]. The density value for each point (it is represented by *D*(*p*)) is calculated by inversing the area of the Voronoi cell assigned to that point [45]. Thus, the trajectory point density is estimated by constructing a Voronoi diagram using trace lines. In Figure 3a, the Voronoi cells corresponding to denser areas are often smaller, while the cells corresponding to sparse areas are often bigger. The trajectory point density change on both side of the road boundary was great. Due to the Voronoi diagram being a dual graph for Delaunay triangulation, the trajectory density change rate can be expressed by the ratio of the two-node density for a Delaunay triangle edge (Figure 3b), and this value is known as the Density Change Rate Index (DCRI). Let *E_i_* denote a triangle edge, the calculation of the DCRI of *E_i_* is as follows:

(1) $$\mathrm{DCRI}\left(E_{i}\right)=\{\begin{array}{l} \frac{D\left(p_{from}\right)}{D\left(p_{to}\right)}\;,D\left(p_{from}\right)\geq D\left(p_{to}\right)\\ \frac{D\left(p_{to}\right)}{D\left(p_{from}\right)\;}\;,otherwise \end{array}$$

where, *p_from_*, *p_to_* are the two nodes of the Delaunay edge *E_i_*, *D(p_from_)* and *D(p_to_)* are the density of two points as shown in the Figure 3b. In Figure 3c, the density difference is small in road areas and nonroad areas, and the DCRI value of a triangle edge is small. Conversely, the density difference is large in the road boundary region, and the DCRI value of the edge is big. Therefore, a triangle edge with bigger DCRI value has a greater possibility of being the road boundary. The triangle edges are divided into two categories through setting a value threshold, and the threshold value is called the *DCRI_Value*. If the DCRI value of a triangle edge is large than or equal to the *DCRI_Value*, the edge can be used as road boundary, as shown in Figure 3c. Otherwise, the edge cannot be used as boundary.

Second, the distance index is explained. For the CDT constructed using trajectory lines in Figure 3d, the length of edge within the road area is short while the length of edge within the non-road area is long. Track points of these long edges can be outliers which are caused by a GPS positioning error [23]. Thus, the edge length not only can be used as a boundary descriptor but also can eliminate outliers, which is called the Distance Index (DI). According to the statistical analysis of Delaunay edge length, an adaptive threshold value which is called *LenValue* is calculated as follows [46]:

(2) LenValue=LenMean(DT)+α×LenVariation(DT)

where *DT* represents the Delaunay triangulation network, *LenMean(DT)* is the mean length of edges in DT, *LenVariation(DT)* is the standard deviation of the length of all edges in DT; and α is an adaptive parameter. In Figure 3d, the triangle edges can be divided into two classes according to the *LenValue*. If the edge length is smaller than the *LenValue* it can be used as a road region, as shown in Figure 3d. When the length is larger than or equal to the *LenValue*, it can be used as road boundary or outlier.

**Figure 4:**
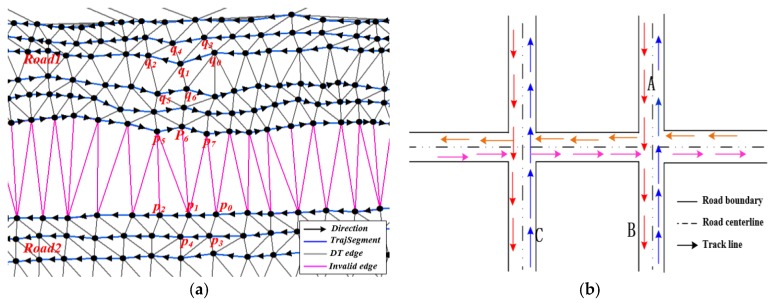
Using movement feature to detect road boundary. (**a**) Classifying edges using vehicle movement direction; (**b**) the road traffic direction.

Third, movement features. The above two descriptors are limited in detecting the boundary due to only considering the geometric features. This paper seeks to integrate movement parameters (e g., direction) into CDT for detecting the boundary. The movement direction (represented by *dir(p_i_)*) of track point *p_i_* is the direction of vector $\vec{p_{i}p_{j}}$. The *i* represents time stamp, and the points *p_i_* and *p_i +_*_1_ are adjacent points with two successive time stamp. Inspired by the work [25], the edges can be classified using the movement parameters:

(3) $$f\left(E_{i}\right)=\cos\left(dir\left(p_{i}\right),dir\left(p_{j}\right)\right)$$

where *p_i_* and *p_j_* are the two nodes of the triangle edge *E_i_*; *dir(p_i_)* and *dir(p_j_)* are the movement direction of the two point *p_i_* and *p_j_* (e.g., the movement direction of *p_1_* is the direction of vector $\vec{p_{1}p_{2}}$ in Figure 4a); *f(E_i_)* is the cosine of the angle between the two directions. When *dir(p_i_)* and *dir(p_j_)* have common directions, it indicates that the two points are on the same road and *f(E_i_)* > 0; When *dir(p_i_)* and *dir(p_j_)* are orthogonal, it indicates that the GPS traces are on intersections where traces cross perpendicularly and *f(E_i_)* = 0. Therefore, when *f(E_i_)* ≥ 0, the triangle edge *E_i_* can be defined as valid edge, such as the edges *p*_0_*p*_1_, *p*_0_*p*_3_, *q*_0_*q*_1_, *q*_0_*q*_3_ in Figure 4a are valid edges. When *dir(p_i_)* and *dir(p_j_)* have opposite directions, it indicates that *f(E_i_)* < 0 and there will be two cases. Case 1 is the two track points, which are on the two lanes of the same road running in opposite directions, and case 2 is the two track points on different roads [25], as shown in Figure 4b. The two cases can be distinguished according to the length of the triangle edge. For case 1, the length of *p_i_p_j_* (or *E_i_*) is less than or equal to twice the length of track segment *p_i_p_i + 1_*, and the triangle edge *p_i_p_j_* (or *E_i_*) can be defined as valid edge; For case 2, the length of *p_i_p_j_* (or *E_i_*) is greater than twice the length of *p_i_p_i +_*_1_, and the triangle edge *p_i_p_j_* (or *E_i_*) can be defined as invalid edge. Figure 4a shows that the edges *q*_0_*q*_6_, *q*_1_*q*_6_, *q*_2_*q*_5_ are valid edges (these edges on the different lanes of the same road running in opposite directions), and the edges *p*_0_*p*_7_, *p*_1_*p*_7_, *p*_1_*p*_6_, *p*_1_*p*_5_ are invalid edges (these edges are on different roads running in opposite directions). Based on the above analysis, the triangle edges can be classified into two groups according to movement features: the valid edge which can be treated as road region and the invalid edge which can be used as boundary.

#### 3.2.2. Road Boundary Detection Model 

**Figure 5:**
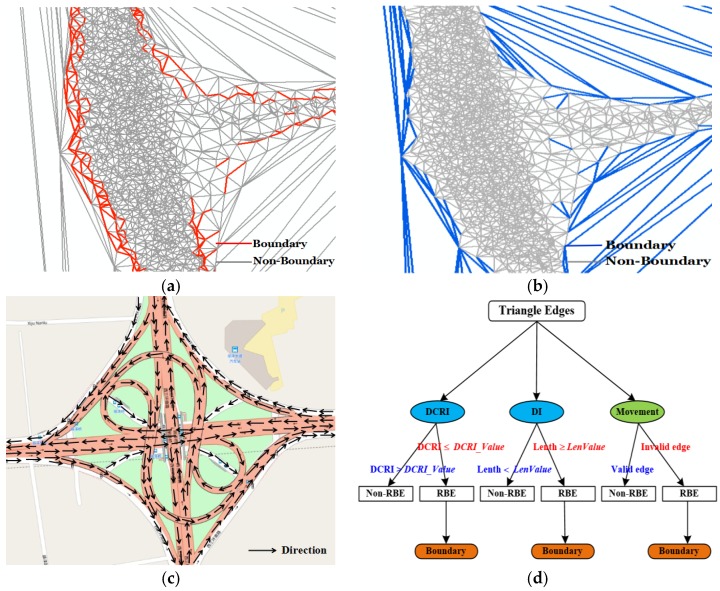
Different descriptors detecting road boundary and road boundary recognition model. (**a**) DCRI detecting the road boundary; (**b**) DI detecting the road boundary; (**c**) movement direction; (**d**) Road Boundary Detection Model.

The three descriptors stated above can be used alone to detect the road boundary. However, each descriptor can partially describe the characteristics of the boundary and identify the road boundary. For example, the road boundary detected by the DCRI descriptor is incomplete, and the topology is disconnected in Figure 5a, especially for the boundary of a branch road with low density track points that cannot be extracted completely. The DCRI descriptor is suitable for the situation where the track point is dense or density changes obviously, such as main roads and road intersections. Fortunately, the DI descriptor is suitable for the trajectory data on the branch road, and combined with movement direction can solve problems that the DCRI descriptor cannot solve. However, only using the DI descriptor makes the extracted boundary become widened, especially for complex structures with high point density such as main roads, overpasses, intersections, and parallel roads, as shown in Figure 5b. Luckily, the DCRI descriptor can get a more accurate boundary for these cases. In Figure 5c, the movement features, such as the direction, can help to detect the boundary more accurately for special environments (e.g., parallel roads, complex road intersections). Therefore, the road boundary detection model is constructed through integrating the three boundary descriptors with CDT for detecting the boundary, as shown in Figure 5d. In the RBDM, for each triangle edge, the edge can be used as the road boundary if it meets one of the following three conditions: the DCRI ≥ *DCRI_Value* or the Length ≥ *LenValue,* or the edge is invalid edge (the three conditions are stated in Section 3.2.1). Thus, the triangle edges are divided into two groups by RBDM in Figure 5d; one is the road boundary edge (RBE) which can be treated as road boundary; another is the non-road boundary edge (non-RBE), which is not a boundary.

### 3.3. Road Boundary Extraction by the Regional Growing Method Based on CDT

#### 3.3.1. Road Boundary Extraction Method

**Figure 6:**
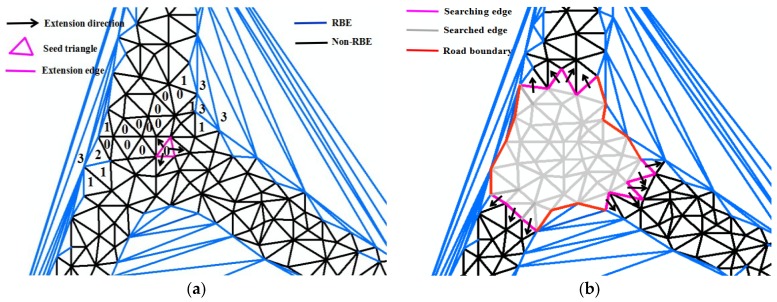
The method of the road boundary extraction. (**a**) Classification of triangles and seed triangle; (**b**) seed point region growing algorithm.

The proposed road boundary extraction method consists of the following steps:

1. Trajectory segments optimization and interpolation after preprocessing GPS point as stated in Section 3.1.
2. Constructing CDT within interpolated trajectory lines.
3. Calculating road boundary descriptors using CDT and the Voronoi diagram as stated in Section 3.2.1, and then constructing the Road Boundary Detection Model, as stated in Section 3.2.2.
4. Classifying Delaunay triangles. The Delaunay triangles are divided into four categories according to the number of RBE, i.e., those having no RBE, one RBE, two RBE, and three RBE are labeled type 0, type 1, type 2 and type 3, respectively, as shown in Figure 6a.
5. Road boundary extraction by the regional growing method. The road boundary extraction algorithm idea is that, under the premise of maintaining the topology connectivity, any type 0 triangle can be used as the seed triangle and uses the seed point region growing algorithm to find the road boundary. As Figure 6a shows, by starting with a triangle connected to the seed point, it is extended to three directions. For a triangle, the search path for one edge entry is two edges of output, meaning that the binary tree width-first search algorithm is used to search the triangulation network. Once the output edge is RBE, the search in this direction of RBE is stopped, as shown in Figure 6b. Therefore, a triangle of type 2 can be regarded as the leaf node with only one edge entered and there is no output. The triangle of type 1 is a non-leaf node with a child node. The triangle of type 0 is a non-leaf node with two child nodes. The triangle of type 3 is the obstacle area, and does not search. In the algorithm design, we adopt the stack data structure to complete the search through the recursive call. The road boundary can be extracted completely by continuing to search according to the adjacency relationship between the edge and the triangle of CDT until all expanding edges are RBE. The road boundary smoothing is performed using the Douglas simplification algorithm.
6. Road centerlines extraction. This involves constructing Delaunay triangulation within the extracted road polygon to extract the road centerlines by the CDT method proposed in [42].

#### 3.3.2. Parameter Selection Method

**Figure 7:**
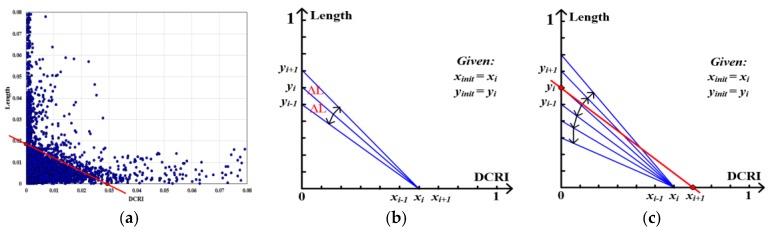
Parameter selection method. (**a**) The triangle edges in Figure 3 is converted into scatter plot taking the normalized DCRI and length values as X and Y axis; (**b**) determining split lines and the initial value; (**c**) parameter automatic searching.

It is important to select the appropriate parameters (*LenValue* and *DCRI_Value*) for the road boundary extraction. Each triangle edge has two attribute values, DCRI value and length value. The edges (Figure 3) are converted into a two-dimensional scatter plot taking the normalized DCRI and Length value as X-axis and Y-axis respectively, as shown in Figure 7a. We can see that the distribution of scatter points (Figure 7a) is aggregated, and this distribution is determined by the distribution of track points. Based on the Section 3.2 analysis, the purpose of the parameter selection is to find the parameter values to divide the edges into two classes: a large number of triangle edges (smaller than *DCRI_Value* and *LenValue*) located in the road region, and a small number of triangle edges (bigger than *DCRI_Value* and *LenValue*) located in the non-road region. If we can find a split line to divide the scatter points into two parts according to parameter value distribution characteristics, then the intersection of the split line and the axis is the parameter value, as Figure 7a shows. The difference of the number of points (DNP) in a local area ∆L (Figure 7b) offset to the split line is used as the standard to select the split line. If the DNP is large, it demonstrates a break in the distribution of the points. Inspired by the work [50], we divide the coordinate axis into m − 1 parts (generating m^2^ split lines), then the DNP of each split line is calculated and the split line with maximum DNP is selected. The parameter automatic search method consists of the following steps:

1. Constructing coordinate system and determining split unit. The triangle edges are converted into a two-dimensional scatter plot taking the normalized DCRI and length values as X and Y axis. The X and Y axis are divided into m−1 parts respectively, then the split points P(*x*) = {*x*_1_, *x*_2_, …, *x_m_*}, P(*y*) = {*y*_1_, *y*_2_,…, *y_m_*}, the local area ∆L and the split lines L(*x*, *y*) = {*x*_1_*y*_1_, *x*_2_*y_1_*, …, *x*_m_*y*_m_, *y*_1_*x*_1_, *y_1_x_2_*, …, *y_m_x_m_*} are obtained, as shown in Figure 7b.
2. Initial search points are set. The two initial search points are set using the nearest split point from the 80th percentile of DCRI and length values, and the initial search points represented by *y_init_* (Y-axis) and *x_init_* (X-axis), as Figure 7b shows.
3. Searching parameter values. For each split point *x_i_* of P(*x*) and the corresponding split lines L(*x_i_*) = {*x_i_y*_1_, *x_i_y*_2_, …, *x_i_y_m_*}, taking split line *x_i_y_init_* as the searching starting-line, the DNP value of each split line (DNP(*x_i_y_i_*)) is calculated by sequence searching in two directions, as Figure 7b shows. For the adjacent two split lines *x_i_y_i_* and *x*_i_*y_i_*_+1_ in the same search direction, when |DNP (*x_i_y_i_*_+1_) − DNP(*x_i_y_i_*)|< ε·N (N is the number of the scatter points, ε is DNP difference ratio of two consecutive iterations, and ε=0.0001 in our paper.) or the all split lines in this direction are searched, the search in this direction is stopped. When the search in the two direction are stopped, the split point x*_i_* has finished searching and starting a new search for next unsearched split point, as shown in Figure 7c. For each split point *y_i_* of P(*y*), also repeat the above operation.
4. Selecting the parameter values. When all split points of P(*x*) and P(*y*) are searched repeat to the Step 3, the average value of two DNP values for each split line is taken as the final DNP value. The split line with maximum DNP is selected, and coordinate value of the two intersection points (the *x_i_*_+1_ and *y_i_* in Figure 7c) of the selected split line and the axis are the parameter values.

**Figure 8:**
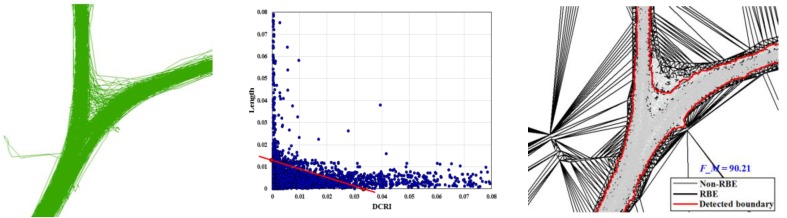

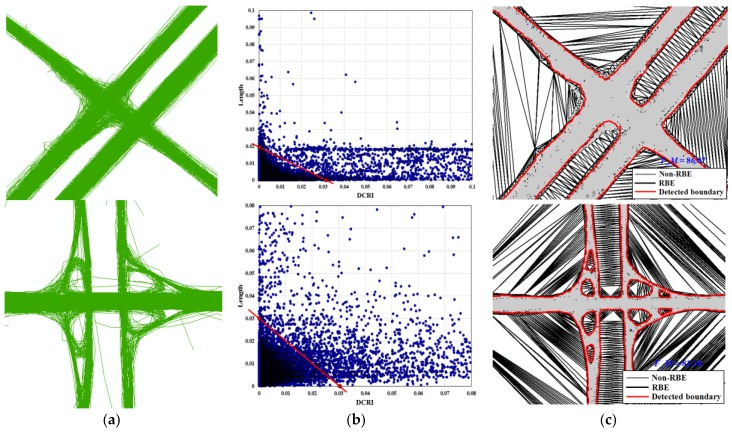
Experimental analysis of parameter values selection. (**a**) Raw GPS trajectory lines; (**b**) parameter values selection; (**c**) road boundary results evaluation.

Based on the above analysis, a set of experiments were conducted to evaluate the parameter automatic selection method (split unit is 0.001 in this work), as shown in Figure 8. In Figure 8, although the road structure and the amount of track data are different, the proposed method can automatically select the appropriate parameters according to the characteristics of the data distribution. For simple road structure in Figure 8a, the F-Measure of the extracted boundary obtained by the proposed method is over 90%. For the complex road structure, the F-Measure of the results can be achieved to 80%. From Figure 8a to Figure 8c, although the amount of track data has significant difference (especially for roads with different grades), the parameter selection method can automatically select the parameters according to the feature of the parameter value distribution to adapt to the change of the amount of the trajectory data.

## 4. Experiment and Evaluation

### 4.1. Experimental Data

**Figure 9:**
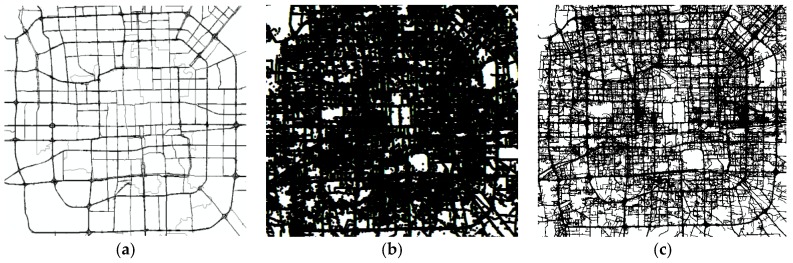
Experimental area and vehicle trajectory data. (**a**) Experimental area; (**b**) trajectory points; (**c**) trajectory lines.

To verify the validity of the proposed approach, vehicle traces from the 12,000 taxis in Beijing were tested, as shown in Figure 9. For the data set of 144,205 GPS traces in one day, however, the sampling frequency ranges from 10 s to 120 s, the average sampling interval is 40 s. The Beijing dataset covers all road types, including curved roads and five-way intersections, with a great degree of uneven distribution in the road network. For the ground truth data, we selected portions of OpenStreetMap (OSM) data and remote sensing images obtained from Tianditu (a public map service released in 2013 by the National Geomatics Center of China) that we manually verified. The road boundary data and road centerline baseline were generated manually using high-resolution satellite imagery. All the experiments are implemented in Visual Studio based on ArcGIS10.2 and Microsoft Windows 8.

### 4.2. Experimental Results

**Figure 10:**
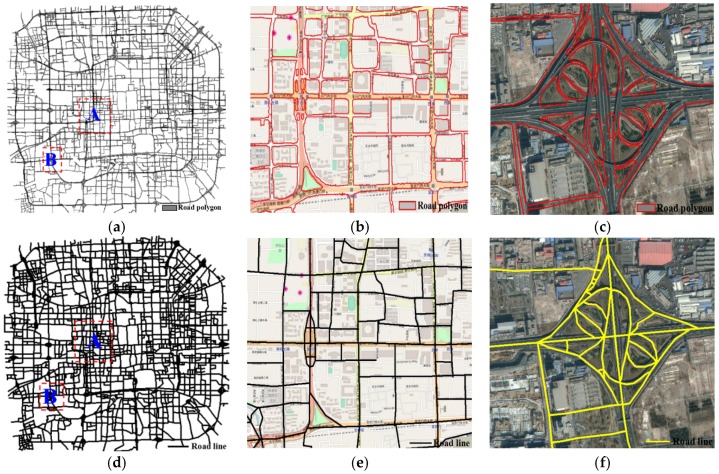
The results of road boundary and road centerlines. (**a**) Road boundary results; (**b**) evaluation with OSM (at A in (**a**)); (**c**) complex intersection (at B in (**a**)); (**d**) road centerline results; (**e**) road line overlaid on OSM (at A in (**d**)); (**f**) complex intersection (at B in (**d**)).

As a conventional method of preprocessing to reduce noisy traces, we removed trace segments with average speeds exceeding 120 km/h and shorter than 15 m [16]. The abnormal trace segments are detected and filtered by the optimization method stated in Section 3.1, then the normal trace segments are interpolated adaptively. The constraint Delaunay triangulation is constructed using one day of interpolated trace lines. The Road Boundary Detection Model is constructed by using the descriptor of DCRI, DI and movement features (selecting parameter values by the method in Section 3.3.2). Using the RBDM to detect the road boundary through classifying the triangle edges, the road boundary is extracted by the seed region growing algorithm, with the results shown in Figure 10a–c. Lastly, Delaunay triangulation is constructed again using the extracted road polygon, and the road centerline is extracted for map construction by the CDT method [42], with the centerline results as shown in Figure 10d–f. The road data results were overlaid on the OpenStreetMap data and remote sensing image. In Figure 10, the overall results of road polygon and road centerline basically cover the road network in the experiment area and with higher accuracy. For the different levels of roads in an urban area, the extracted road boundary (Figure 10b) and centerline with high precision (Figure 10e). For the complex road structures (e.g., overpass, parallel road), our method can distinguish the road area from the non-road area (Figure 10c). Meanwhile, the proposed approach is suitable for the density disparity of track points in different hierarchies of road networks (Figure 10b,e). As to the extracted boundary with high accuracy, the extracted road centerlines can maintain the correctness of topology (Figure 10f). However, part of the low-grade road boundary cannot be extracted or the results have low accuracy because of those roads with few track lines.

### 4.3. Results and Evaluation

To evaluate the proposed method, based on the baseline road vector data of Beijing, the results of our method are compared with the results of the KDE method [37] and DT method [19,42]. Results of the road boundary are evaluated by the polygon overlaying method, and the results of road centerlines are evaluated by the buffer matching method [14,26]. There are three indexes including precision (*P*), recall (*R*) and *F*-measure (*F*) that are computed to evaluate the results quantitatively, and which are defined according to the paper [14]:

(4) $$P=\frac{matched}{matched+spurious}$$

(5) $$R=\frac{matched}{matched+empty}$$

(6) $$F=\frac{2\times P\times R}{P+R}$$

*matched* is the extracted results which matching the reference data, while *spurious* is the extracted results which cannot match the reference data, *empty* is the parts of the ground truth data which is missing in the extracted data.

#### 4.3.1. Comparison of Our Results with the KDE and DT method

**Figure 11:**
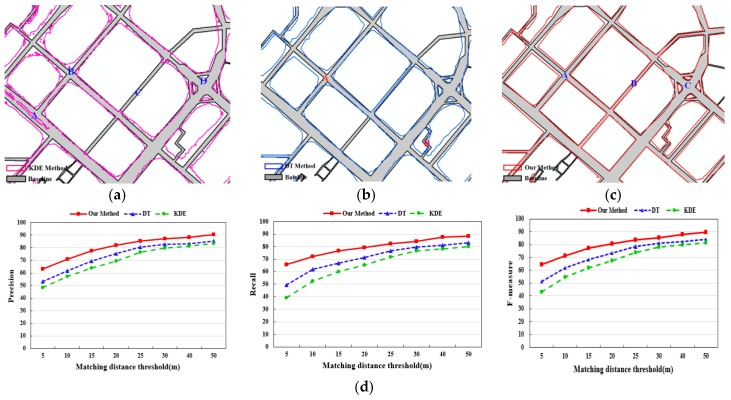
Comparative evaluation of experimental results of three methods. (**a**) Results of the KDE method (partial); (**b**) results of the DT method (partial); (**c**) results of the proposed method (partial); (**d**) evaluation of accuracy of road centerlines extracted from the extracted polygons.

As a qualitative evaluation of the results, the extracted road boundary was overlaid on the baseline data of the corresponding area (partial results), as shown in Figure 11a–c. It is clear by visual inspection that the accuracy and coverage of the road boundary extracted by the proposed method are higher than the compared methods. The KDE method, however, produced messy outputs where the road had high density GPS points (at B in Figure 11a), and the road boundary with low track lines cannot be extracted (at A and C in Figure 11a). The extracted boundary by DT method was wider than the real boundary due to not filtering the abnormal trajectory segments generated by the low sampling rate and only using the length of the Delaunay edge to detect the road boundary, especially for road intersections, as A and B in Figure 11b shows. Certainly, due to the GPS positioning error, the results of the three methods are difficult to coincide with the real boundary (Figure 11). Moreover, the coverage of the boundary was limited due to the trace coverage. As such, streets inside Chinese residential areas are less motorized and subsequently missing from the vehicle tracking data. 

Furthermore, quantitative evaluation results of the three methods are presented in Table 1 and Figure 11d. According to Table 1, the road boundary extracted by our method can be improved by more than 10% in accuracy and completeness compared with the KDE and DT method. The road centerlines are extracted by the CDT method of [42] from the extracted boundary of the three methods. In Figure 11d, the accuracy of the road centerline extracted from the road polygon by our method in the 5 m buffer has improved 8% compared with the other two methods.

#### 4.3.2. Comparison with the Experimental Results under Different Situations

**Figure 12:**
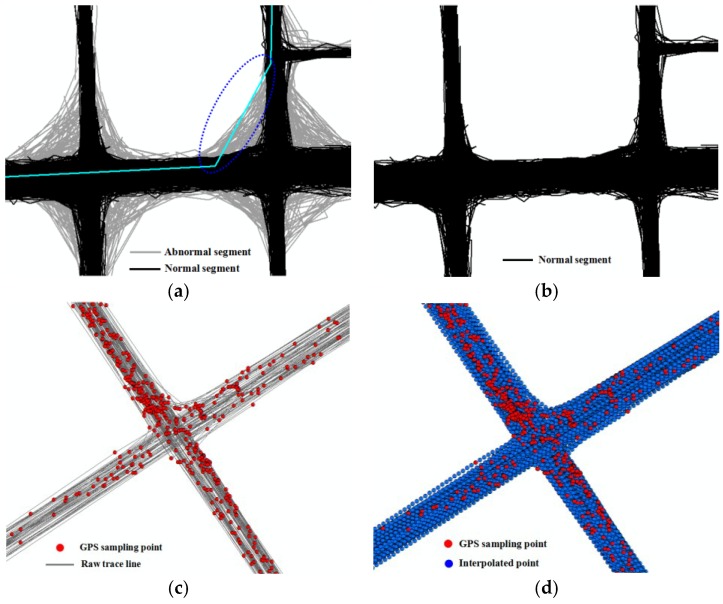
Trace segments optimization and interpolation. (**a**) Abnormal trace segments detection; (**b**) trace segments optimization; (**c**) trace lines with low sampling rate; (**d**) trace lines interpolation adaptive.

Compared with the KDE method and DT method, the proposed method has higher coverage and accuracy, the reason for which lies in the following three aspects:

Firstly, trace segments optimization and interpolation. Due to the sparse sampling rate of vehicle traces, a large number of abnormal trace segments are generated at the corners of road when generating one trace for each vehicle by connecting its consecutive samples, as shown in Figure 12a. In our method, those abnormal trace segments are detected and filtered by the optimization method stated in Section 3.1 to eliminate the negative influence of messy trajectory portions and improve the accuracy of results, and the optimization results are shown in Figure 12b. In Figure 12c, although the total amount of the traces is huge, the GPS points are not dense enough for extracting the road boundary because the sampling rates for a single trace are sparse. Therefore, the optimization trace segments are interpolated by the method described in Section 3.1 to ensure there are enough GPS points to improve the completeness of the boundary, as shown in Figure 12d.

**Figure 13:**
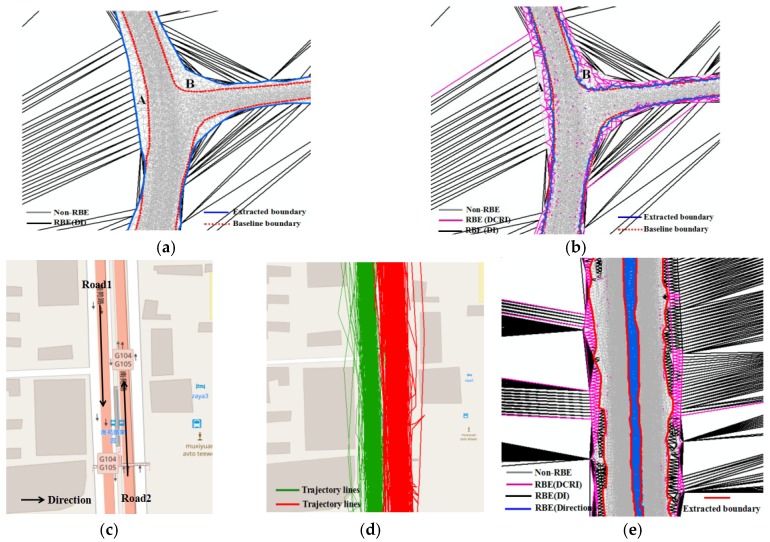
Advantages of the road boundary detection model compared to the DT method. (**a**) Length of Delaunay edge descriptor (DT method); (**b**) boundary detection model (proposed method); (**c**) different roads close in space; (**d**) traces on different road; (**e**) boundary detecting by RBDM.

Secondly, constructing the Road Boundary Detection Model. In this work, the RBDM is constructed by CDT and the Voronoi diagram. The RBDM uses the area of the Vonoroi cell to present the local density distribution of track points, and the length of the Delaunay edge presents the distance of neighbor points. In Figure 13a, the extractions of road polygons by the DT method [19,42] are wider than the real road area, especially for the road with huge track points, such as the road intersection (at A and B in Figure 13a), or main road. The reason for this is that the DT method only uses the length of the Delaunay edge to detect the road boundary but ignores the role of the track point density change rate. Compared with the DT method, the RBDM in our method uses the DCRI descriptor to eliminate the error boundary (at A and B in Figure 13b) and improve the result precision. Figure 13b shows that the extracted boundary by our method is close to the real boundary. Moreover, the detection model combines trace features with the CDT to detect the boundary in special cases, such as an adjacent road in space and the boundary of different lanes. In Figure 13c–d, although the two roads are very close in space and the tracks on different roads overlap, our method can detect and extract the boundary of different roads through using the trajectory direction to classify Delaunay edges by the RBDM (Figure 13e). Despite this success, it was difficult to discriminate adjacent roads or lanes that had the same direction but were different roads.

**Figure 14:**
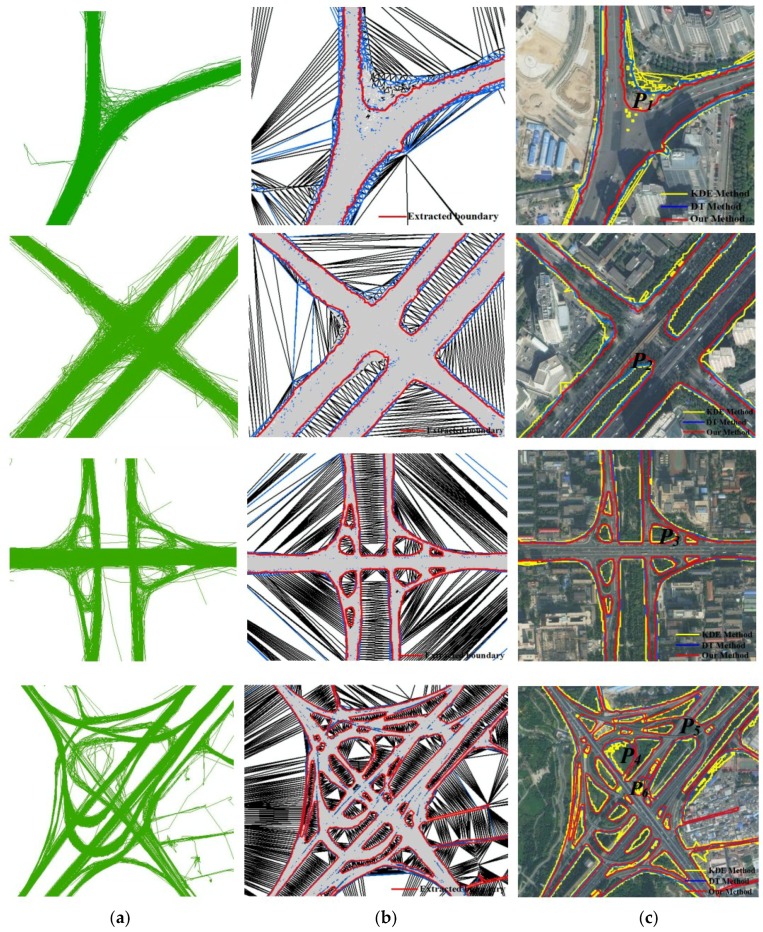
Road boundary detection under different road structures by the proposed approach compared with KDE and DT methods through overlaying with a Google image for the study area. (**a**) Raw GPS traces; (**b**) boundary detection and extraction; (**c**) comparative evaluation.

In addition, our proposed approach was able to detect and extract the road boundary of various types of road structures, including crossroads, T-junctions, double trunk roads, and roundabouts. After overlapping the road boundary with a Google image (Figure 14), we observed the boundary with higher accuracy and coverage. Figure 14 shows that the boundary of *p_1_* and *p_2_* extracted by our method is more accurate than the compared methods due to considering the track point’s density distribution difference. Moreover, the ramps and approach bridges in the complex road intersection can be distinguished from the non-road area (*p_3_* and *p_4_* in Figure 14) by our method. The boundary of the ramp *p_4_* also can be detected and extracted through interpolating the track lines, while the KDE method cannot do it. In terms of topological correctness, the KDE method has more isolated road polygons and the line topology breaks and the road boundary or road centerline follow a zig-zag pattern, while the proposed method can solve these problems.

Although this was successful, the proposed approach displayed certain problems in the processing of boundary extraction. For example, despite the results of our method achieving higher accuracy than the compared methods, the results of the three methods are difficult to coincide with the real boundary. In Figure 14, the boundary error data is generated at *p_2_* and *p_3_* by the three methods due to GPS positioning errors. *p_5_* in Figure 14 was identified as a road area that is a non-road area where, actually, three roads crossed over each other with a large number of track lines leading to track points error accumulation. *p_6_* in Figure 14 was identified as a non-road area that is actually a road area because only six traces passed, and some tracking segments were eliminated as abnormal segments. In the future, we will consider solving these problems using other methods.

**Figure 15:**
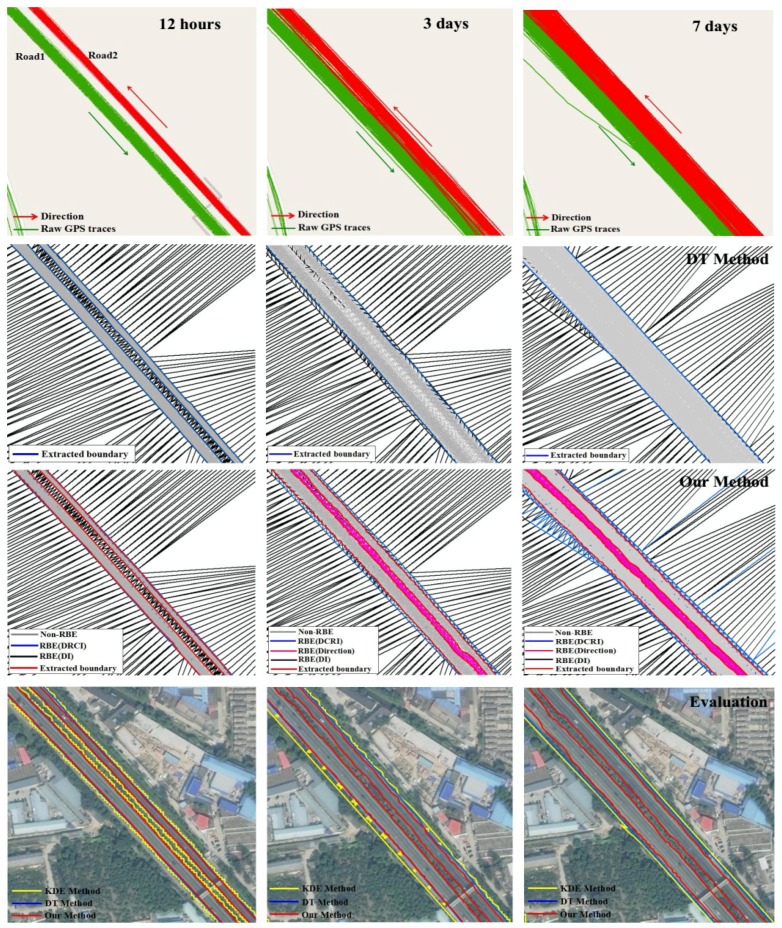
Comparison and analysis of road boundary extraction for various time spans.

Finally, GPS traces with various time span are considered. Quantitative and density differences of track points at different time span add to the fuzziness of the boundary, especially for roads in closely spaced. Taking Beijing-Tianjin Expressway for example, the trajectory lines of 12 h (3181 points), 3 days (30,180 points) and 7 days (71,869 points) respectively are used to conduct the experiment, as Table 2 and Figure 15 shown. The extraction of the boundary from tracking lines at different time span by the KDE and DT method have two main issues. One is the road boundary imprecision. In Figure 15, for the 3 days and 7 days track lines, the extracted boundary by the KDE and DT method are wider than the real boundary. In particular, the non-road area outside the road boundary is identified as road area by the KDE method. As Table 2 shows, recall and precision initially increase rapidly as the number of trace increases. As time increases, increasing the amount of GPS points make the precision of KDE and DT decrease quickly. Luckily, the RBDM can adapt to the change of the amount of the track data using the parameter automatic selection method. In Table 2 and Figure 15, the boundary precision of our method is higher than the two methods, especially for the 7 days track lines. Another issue is that the roads which are closer in space cannot be distinguished. In Figure 15, the track lines will cover the non-road areas between the two roads as time goes on. For the 3 days and 7 days track lines, the KDE and DT method merge the two roads into one road due to these methods not considering the movement features (Figure 15), which generates error data and reduces the accuracy of the data. The problem can be solved by our method through combining track direction into the boundary detection model. In Figure 15, road area and non-road area can be distinguished accurately by the proposed method to improve the boundary precision.

#### 4.3.3. Comparison with Methods Based on Remote Sensing Image

**Figure 16:**
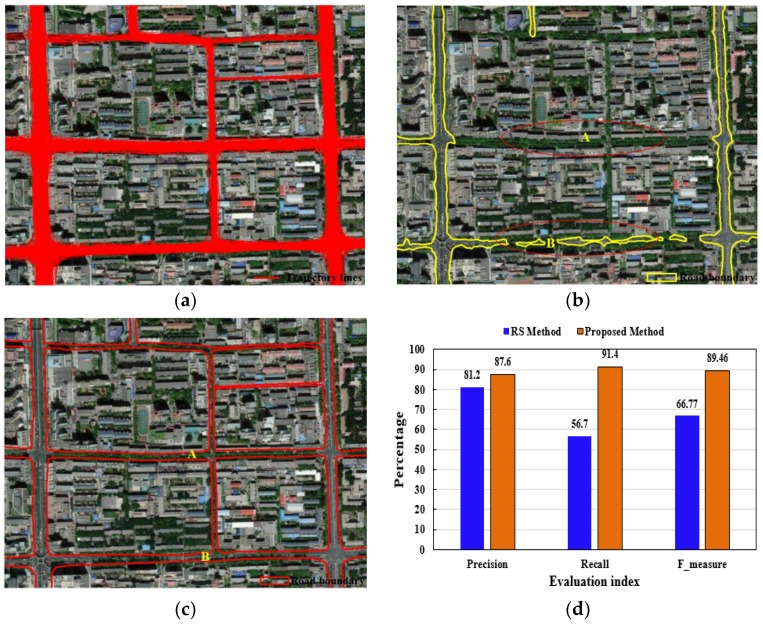
Comparison of the results of the proposed method with the remote sensing morphology method. (**a**) Raw track lines and remote image; (**b**) boundary extracted from remote sensing; (**c**) boundary extracted by the proposed method; (**d**) evaluation of road boundary results.

Taking the Hepingli community in Beijing as a case, we used the morphology method to extract the road boundary from remote sensing image, as shown in Figure 16. Comparison of the two methods show that our method outperforms the remote sensing method in the following aspects:

- First, given the fact there are many shelters such as trees, tall buildings, the extracted road boundary using remote sensing image is not complete and has many low-precision or error data. In Figure 16b, remote sensing method can’t extract these low-grade roads with many trees, and the extracted data with low-precision such as topology errors (at B in Figure 16b). The recall and precision of our method are higher than the remote sensing method in Figure 16d.
- Second, the extraction of traffic flow semantic information from GPS tracks. Compared with remote sensing image, track lines can also extract semantic features such as traffic flows, traffic rules, and spatio-temporal behavior [23,24,25,26,27,28]. These traffic semantic information can help to identify and extract the road data, such as extracting different lanes, detecting road changes. Certainly, the two methods have their advantages and disadvantages, the road data (geometry and semantic) is extracted by fusing the high-resolution aerial images and GPS track data to solve problems which are caused by using a single data [21].

### 4.4. Discussion

As our evaluation results indicate, the proposed method was validated as an effective approach for taking low-frequency track lines as input for extracting the road boundary and constructing street maps. However, the following problems are worth discussion:

- First, how many track points (track lines) are on a road suitable for road boundary extraction is an uncertain problem. For a road, factors such as road grade (e.g., main road, urban branch), road structure (e.g., road intersection, roundabouts, overpass with ramps), vehicle frequency, traffic flow, track sampling frequency, time interval and more affect the number of track points. Not only does a single factor affect the quantity of data, but also the combination of different factors has different effects on it. Although this paper uses the interpolation method and RBDM to solve some problems stated in Section 4.3.1, there still needs to be consideration of more factors to solve the problem by quantitative modeling.
- Second, GPS positioning noise (error or uncertainty) is a problem. Taxi GPS traces also show that the positions of commodity GPS are noisy and inaccurate (position accuracy is about 10–15 m) [23]. Especially, the distribution of GPS positioning error has spatial heterogeneity in the road environment, it is also affected by many factors such as device, weather, environment (buildings, trees). To accurately measure the GPS positioning error in the road network environment, we need to combine the multisource data (photos, street view data, etc.) to evaluate the friendliness of road environment to GPS, and the GPS positioning error estimation model is established for different road geographical environment. This requires lots of work and a perfect model. Therefore, the GPS error estimation in the road environment and the influence of GPS error on road data extraction are the research works in the future.
- Third, multisource data fusion is needed. When only using a single taxi trajectory data to extract the road boundary, its accuracy and coverage need to be improved, especially for the residential areas and urban branches. As stated in Section 4.3.2, multisource data fusion (e.g., multi-type VGI data, traditional data and big data) is an important method to consider for road information acquisition and urban sensing.

## 5. Conclusions

In this paper, an approach for extracting road boundary information from crowdsourcing low-frequency vehicle trajectory data is proposed based on constraint Delaunay triangulation. The method was validated and evaluated using taxi GPS data with a sampling interval of approximately 40 s in Beijing. The results indicate that the proposed method improves precision and coverage compared with the KDE and DT method. It was demonstrated to be useful for quality enhancement in low sampling rate situations when handling the vehicle tracking lines of different road structures and different time intervals in a feasible way.

Furthermore, there were still some problems that should be tackled to improve the usability of the proposed method. First, we need further research on the road boundary or road centerlines of three-dimensional complex road intersections and overpasses, especially for complex conditions such as urban canyons and low-grade roads. The precision and coverage of boundary results should be raised through improving the RBDM and fusing multisource data. In addition, trajectory data as the VGI data or crowdsourced data, it is important to be aware of the quality and reliability of VGI data and mult-source crowdsourcing data [7]. Second, this work only extracts the geometry data of roads, however it also needs to extract road attributions such as road names, lanes, and traffic rules.

## Figures and Tables

**Table 1:** The evaluation of road boundary results of three methods.

	Precision	Recall	*F*_Measure
KDE Method	66.1	58.7	62.18
DT Method	78.3	70.6	74.25
Our Method	88.6	80.2	84.19

**Table 2:** The quantitative evaluation of road boundary results in Figure 15.

-	12 Hour			3 Days			7 Days		
	Precision	Recall	*F*_m	Precision	Recall	*F*_m	Precision	Recall	*F*_m
KDE method	92.15	89.78	90.94	68.71	100	81.45	45.18	100	62.23
DT method	94.34	85.65	89.78	71.62	98.61	82.97	64.25	100	78.23
Our method	95.68	86.21	90.69	91.27	96.24	93.68	90.19	99.3	94.56
